# Ensuring a safe, tobacco free future for the young: protecting children from tobacco industry interference

**DOI:** 10.1136/tc-2024-058698

**Published:** 2024-04-19

**Authors:** Mary Assunta, Phil Chamberlain

**Affiliations:** 1 Southeast Asia Tobacco Control Alliance, Bangkok, Thailand; 2 Advocacy at Global Center for Good Governance in Tobacco Control, Bangkok, Thailand; 3 Tobacco Control Research Group at the University of Bath, Bath, UK

**Keywords:** Addiction, Advertising and Promotion, Advocacy

The tobacco industry needs to replace the eight million people who use tobacco^1^ and die every year from tobacco-related diseases. These are often recruited among the young, since 9 out of 10 people who smoke start before the age of 18.^2^ Young customers keep the industry viable. The WHO estimates over 40 million children aged 13–15 years use tobacco.^3^ While most governments have implemented measures to protect minors from tobacco use in accordance with the WHO Framework Convention on Tobacco Control (FCTC), the fight to protect the young remains intense as the tobacco industry continues to target youth. The tobacco industry is ‘inordinately profitable’^4^; the profits of the six largest tobacco companies in 2018 were US$55 billion, more than Coca Cola, PepsiCo, Nestle, Mondelez, FedEx, General Mills, Starbucks, Heineken and Carlsberg combined.^5 6^ In the USA alone, in 2019, the largest tobacco companies reportedly spent US$8.2 billion marketing cigarettes and smokeless tobacco.^7^

As the world marks World No Tobacco Day on 31 May, this year’s theme, ‘protecting children from tobacco industry interference’, is both timely and pertinent.

Transnational tobacco companies have been repeatedly exposed for their deliberate tactics targeting youth despite bans on all forms of tobacco advertising, promotion and sponsorship (TAPS). The Truth Initiative’s fifth report, *While You Were Streaming: Tobacco’s Starring Role*,^8^ on social media advertising in 2021 found only limited improvement in reducing young people’s exposure to smoking imagery. According to the report, 60% of the 15 most popular shows among people aged 15–24 years in the USA contained depictions of tobacco. Nearly half (47%) of the top films released in 2021, including 17 PG-13 rated or under movies, featured tobacco imagery, according to a review by the University of Chicago’s National Opinion Research Center.^9^ An analysis of the top 2021 Billboard songs revealed that 12.8% of songs had 290 tobacco depictions in their music videos and were viewed over two billion times.^8^ That at least was down significantly from 23% in 2020.

Two tobacco giants targeted more than 60 countries with expansive marketing campaigns for Velo, a nicotine pouch and Vuse, an e-cigarette, from British American Tobacco (BAT), and IQOS, the heated tobacco product from Philip Morris International (PMI).^10^ 40% of the audience engaging with BAT and Philip Morris marketing content on social media are young people under 25 years, according to a Campaign for Tobacco-Free Kids study.^11^ A report from STOP^12^ outlines 10 examples of how transnational tobacco companies target young people such as sponsoring music festivals, using influencers who look under 25 to promote its products on social media and failing to add mandatory health warnings.

Formula 1 sponsorship is one clear example of how the industry is getting around existing TAPS rules. PMI and BAT have spent billions of dollars over the years in sponsorship deals with Formula 1 racing teams.^13^ As the sport looked to expand into new markets and attract a young and more female demographic, this has played perfectly into the hands of tobacco companies. The recent link up with Netflix and the Drive to Survive series has added an extra layer of free visibility to tobacco firms. Liberty Media, which owns Formula 1, gave Netflix full access to the sport to create a popular fly-on-the-wall series. That also means more exposure for tobacco company messaging on Ferrari and McLaren livery. Researchers from Formula Money looked at 416 min of footage from Season 4 and found just over a third (35.8%) contained images from cigarette companies, meaning that Netflix has likely screened a billion minutes of free tobacco branding since 2019. Viewer demographics are difficult to gauge but Drive to Survive swings towards an under-25 market with a relatively even gender viewership. Only 41% of Drive to Survive viewers also watched the first 3 weeks of Formula 1 races in 2022, so the series meant tobacco firms reached many new consumers.^14^

This approach is not surprising given the long history within the industry of targeting new markets and new products. From marketing ‘light’ or ‘mild’ cigarettes in the 1970s,^15^ giving the impression these are less harmful and promoting menthol cigarettes—which mask the harshness and dangers of smoking—to women, the black community and teens. Other tactics to attract young people include using flavours in cigarillos^16 17^ and e-cigarettes^18^ and offering discounts and promoting positive experiences on social media.^19^

Attempts to de-glamorise tobacco products and protect young people through measures such as prominent pictorial warnings and plain packaging have been met with tobacco industry interference, including taking governments to court. For example, Australia’s plain packaging legislation, an effective tobacco control measure to reduce the appeal of tobacco and discourage smoking uptake, especially among young people,^20^ was (unsuccessfully) challenged by several transnational tobacco companies in four separate cases claiming it was unconstitutional.^21^ In the Philippines, Balanga City was sued by the Philippines Tobacco Institute for its Ordinance No 09 of 2016 that banned the sale, distribution, use, advertising and promotion of tobacco products and/or Electronic Nicotine Delivery Systems within 3 km of the university town and for its Tobacco-Free Generation Ordinance of 2016 that regulated the sale of tobacco products to those born on or after 1 January 2000.^22 23^ In Sri Lanka, BAT’s local subsidiary sued the Health Minister for gazetting pictorial health warnings to cover 80% of cigarette packs, arguing it violated their intellectual property rights. The court ruled in favour of the industry, recommending that warnings should be reduced to 50%–60% of the pack surface.^24 25^ In Thailand, transnational tobacco companies unsuccessfully sued the Minister of Public Health to prevent the size of pictorial health warnings being increased to 85%.^26^

But young people are not remaining passive targets. At the 10th session of the WHO FCTC Conference of the Parties (COP10), youth were a significant civil society presence. Global Youth Voices, a movement of youth organisations across 130 countries, took the floor and made an earnest plea to the governments to remember “their children, nieces and nephews when making decisions at COP 10, and to choose to be remembered as the ones that protected them from the harms of tobacco.”^27^ It was an important reminder that industry interference would impact down the ages and a powerful counter to attempts at tobacco industry interference in WHO FCTC implementation, including at Conference of the Parties sessions.^28–30^

It is vital that the industry’s targeting of young people is resisted. Fortunately, the WHO FCTC has the tools to do this. COP10 adopted specific guidelines to address cross-border advertising of tobacco and nicotine products online and geared towards youth.^31^ Parties to the WHO FCTC should enforce Article 13^32^ that calls for a comprehensive ban on all TAPS. The media platforms which enable the tobacco companies to get their message to young people need to reject the industry. Governments need to collaborate to regulate both tobacco companies and the platforms which carry their messages. The setting of health policies should be protected as Article 5.3 states to ensure no undue industry interference.^33 34^

The need to protect young people and future generations is everyone’s responsibility, as amply demonstrated in this year’s World No Tobacco Day theme.
